# Gasotransmitters for the Therapeutic Prevention of Hypertension and Kidney Disease

**DOI:** 10.3390/ijms22157808

**Published:** 2021-07-21

**Authors:** Chien-Ning Hsu, You-Lin Tain

**Affiliations:** 1 Department of Pharmacy, Kaohsiung Chang Gung Memorial Hospital, Kaohsiung 833, Taiwan; cnhsu@cgmh.org.tw; 2 School of Pharmacy, Kaohsiung Medical University, Kaohsiung 807, Taiwan; 3 Department of Pediatrics, Kaohsiung Chang Gung Memorial Hospital and Chang Gung University College of Medicine, Kaohsiung 833, Taiwan; 4 Institute for Translational Research in Biomedicine, College of Medicine, Kaohsiung Chang Gung Memorial Hospital and Chang Gung University, Kaohsiung 833, Taiwan

**Keywords:** kidney disease, gasotransmitter, carbon monoxide, hypertension, developmental origins of health and disease (DOHaD), hydrogen sulfide, asymmetric dimethylarginine, heme oxygenase, nitric oxide

## Abstract

Nitric oxide (NO), carbon monoxide (CO), and hydrogen sulfide (H_2_S), three major gasotransmitters, are involved in pleiotropic biofunctions. Research on their roles in hypertension and kidney disease has greatly expanded recently. The developing kidney can be programmed by various adverse in utero conditions by so-called renal programming, giving rise to hypertension and kidney disease in adulthood. Accordingly, early gasotransmitter-based interventions may have therapeutic potential to revoke programming processes, subsequently preventing hypertension and kidney disease of developmental origins. In this review, we describe the current knowledge of NO, CO, and H_2_S implicated in pregnancy, including in physiological and pathophysiological processes, highlighting their key roles in hypertension and kidney disease. We summarize current evidence of gasotransmitter-based interventions for prevention of hypertension and kidney disease in animal models. Continued study is required to assess the interplay among the gasotransmitters NO, CO, and H_2_S and renal programming, as well as a greater focus on further clinical translation.

## 1. Introduction

Gasotransmitters, such as nitric oxide (NO), carbon monoxide (CO), and hydrogen sulfide (H_2_S), are small gaseous molecules that penetrate membranes and play key roles in biology. Although these gases are toxic in excess, they are endogenously generated and exert specific biological functions at the physiological level [1,2,3]. A brief overview of their toxic and physiological levels is given in Table 1 [1,2,3]. Since it was identified as the endothelium-derived relaxing factor in the 1980s, NO has rapidly gained attention as one of the most important signaling molecules in the cardiovascular system [4]. A decade later, CO emerged as a gaseous vascular modulator of the cardiovascular system [5]. H_2_S, next to NO and CO, has emerged as a third gasotransmitter with key roles in the regulation of cardiovascular and other systems [6]. All three gases have a significant impact on human health and potential value as a therapeutic target [1,2,4].

Chronic kidney disease (CKD) and hypertension are major non-communicable diseases, which are the leading causes of global deaths. According to the WHO, one in five women and one in four men have hypertension worldwide [7]. An estimated ~10% of the global population has CKD [8]. Hypertension and CKD are closely associated with an overlapping and interlinked cause and effect relationship [9], such that hypertension can lead to CKD progression and CKD is the most common cause of secondary hypertension. Of note, hypertension as well as kidney disease can take their origins in early life, and when identified early, can be healed to prevent more associated disorders and serious complications.

During kidney development, various early-life adverse environmental conditions can lead to hypertension and kidney disease in adulthood [10]. The idea was recently named “Developmental Origins of Health and Disease” (DOHaD) [11]. Conversely, through shifting therapeutic approach from adulthood to early life, namely, reprogramming, we have the potential to revoke disease processes before disease becomes apparent [12,13].

The three gases and their roles in established kidney disease and hypertension have been extensively reviewed elsewhere [4,5,6,14,15,16,17]. However, evaluating their impacts on hypertension and kidney disease of developmental origins has not been sufficiently addressed [18,19]. The aim of this review is to discuss, within the limits of present knowledge, how the three gasotransmitters are implicated in the developmental programming of hypertension and kidney disease. In particular, the review focuses on the potential of gasotransmitters for therapeutic prevention against hypertension and kidney disease of developmental origins.

We searched the PubMed/MEDLINE databases for studies published in English using the following search terms: “gasotransmitter”, “kidney disease’, “developmental programming”, “DOHaD”, “nitric oxide”, “hydrogen sulfide”, “carbon monoxide”, “heme oxygenase”, “oxidative stress”, “nephron”, “nephrogenesis”, “mother”, “pregnancy”, “gestation”, “offspring”, “progeny”, “reprogramming”, and “hypertension”. We also used the reference lists of identified articles to find additional studies. The last search was made on 30 May 2021.

## 2. Implications of Gasotransmitters in Pregnancy

**Figure 1:**
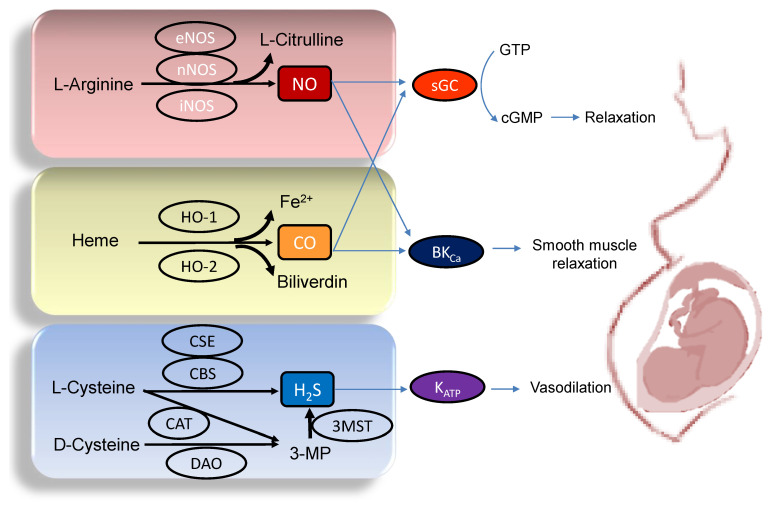
Schematic illustration of the enzymatic synthesis of NO, CO, and H_2_S and downstream signaling able to maintain maternal and feto-placental homeostasis. NO (upper panel) is formed by neuronal NOS (nNOS), endothelial NOS (eNOS), and inducible NOS (iNOS) from L-Arginine. Heme oxygenase-1 (HO-1) and -2 (HO-2) enzymes degrade heme to generate CO, iron, and biliverdin (middle panel). Three enzymes have been identified to enzymatically generate H_2_S (Lower panel), cystathionine β-synthase (CBS), cystathionine γ-lyase (CSE), and 3-mercaptopyruvate sulphurtransferase (3MST). CBS and CSE produce H_2_S using L-cysteine. In an alternative pathway, 3-mercaptopyruvate (3-MP), the substrate for 3MST to produce H_2_S, is provided by cysteine aminotransferase (CAT) using L-Cysteine and D-amino acid oxidase (DAO) using D-Cysteine, respectively. The blue arrow lines indicate downstream signals of gasotransmitters in the maintenance of homeostasis in pregnancy. NO and CO both can activate soluble guanylate cyclase (sGC) to increase cGMP, resulting in smooth muscle relaxation. The large-conductance Ca^2+^-activated K^+^ channel (BK_Ca_) can also be regulated by NO and CO to elicit vasodilatation. Additionally, through the activation of ATP-sensitive K^+^-channels (K_ATP_), H_2_S can cause vasodilation in pregnancy.

A variety of adverse conditions during pregnancy can affect fetal development resulting in hypertension and kidney disease in adult offspring, including maternal malnutrition, maternal exposure to environmental chemicals/toxins, maternal illnesses, medication uses in pregnancy, etc. [10,12,13,20,21,22]. Gasotransmitters play a crucial role in the regulation of maternal hemodynamics, placenta vascular development, embryogenesis, feto-placental vascular reactivity, and fetal development during pregnancy [23,24,25]. Abnormalities of gasotransmitter production and signaling in compromised pregnancy are linked to adverse pregnancy and fetal outcomes. A drawing schematic summarizing the enzymatic production of NO, CO, and H_2_S, and signaling pathways able to maintain normal pregnancy and fetal development are depicted in Figure 1. Each gasotransmitter is discussed in turn.

### 2.1. Nitric Oxide

NO plays a vital role in the regulation of feto-placental circulation, fetal development, and transfer of nutrients from mother to fetus in normal pregnancy [26]. NO can be produced by L-Arginine–nitric oxide synthase (NOS)-dependent or NOS-independent pathways. There are three NOSs, namely, neuronal NOS (nNOS), endothelial NOS (eNOS), and inducible NOS (iNOS), which converts L-Arginine to L-Citrulline and generate NO (Figure 1). The NOS-independent pathway involves the reduction of nitrite to NO [27]. This nitrate–nitrite–NO pathway is considered as an alternative source of NO to the classical L-Arginine–NOS pathway. NO bioavailability mainly depends on intracellular L-Arginine concentrations [28]. There are two L-Arginine derivatives, asymmetric and symmetric dimethylarginine (ADMA and SDMA), which share common cationic amino acid transporters (CATs) with L-Arginine to move in and out of cells [29]. Both ADMA and SDMA can compete with L-arginine and inhibit NO production [29,30]. These two methylarginines are formed by a family of protein arginine methyltransferases (PRMTs) [29]. Dimethylarginine dimethylaminohydrolase-1 (DDAH-1) and -2 (DDAH-2) can metabolize ADMA to L-Citrulline and dimethylamine. In early pregnancy, the increase in NO and the concomitant reduction in ADMA assist hemodynamic adaptation and uterine relaxation, to avoid disturbed intrauterine growth of the fetus. Conversely, NO-induced relaxation of the uterus in late pregnancy can be antagonized by physiologically increased ADMA levels to aid in preparing the uterine muscle fibers for the higher contractile activity that is required for successful delivery [31]. NO regulates the relaxation of vascular smooth muscle cells primarily by driving soluble guanylyl cyclase (sGC) to produce cyclic guanosine monophosphate (cGMP). Besides, NO-induced relaxation of human placental arteries is partly mediated through a direct effect on the large-conductance Ca^2+^-activated K^+^ channel (BK_Ca_) [32].

Maternal plasma arginine levels were reduced in pregnancies complicated by intrauterine growth retardation (IUGR) [33]. Likewise, plasma arginine concentrations and placental eNOS abundance were decreased in women with preeclampsia [34]. Conversely, high ADMA levels in pregnant women are associated with preeclampsia [35], gestational diabetes mellitus [36], and fetal mortality [37]. Another line of evidence supporting NO deficiency in pregnancy attributed to adverse maternal and offspring outcome is from animal research. A previous report demonstrated that eNOS knockout pregnant mice displayed uteroplacental hypoxia, resulting in IUGR [38]. Additionally, adult rat offspring born of dams exposed to the L-N^G^-Nitro arginine methyl ester (L-NAME, a NOS inhibitor) in pregnancy developed hypertension, proteinuria, and kidney disease [39,40].

### 2.2. Carbon Monoxide

Like NO, CO is a diatomic low molecular weight gas with similar molecular size and structure [41]. However, CO is a relatively non-radical, chemically stable gas. CO is produced endogenously as a by-product of heme degradation catalyzed by the action of heme oxygenase-1 (HO-1) or -2 (HO-2) enzymes. The two known CO signaling mechanisms are the cGMP-dependent and -independent pathways (Figure 1). Classical CO signaling is similar to NO signaling: CO activates sGC to increase cGMP stimulation of protein kinase G (PKG), resulting in smooth muscle relaxation. Although CO and NO bind sGC with similar affinity, NO-sGC is approximately 25–50 times more active than CO-sGC [42]. Besides, CO can directly enhance the activity of BK_Ca_ in rat vascular smooth muscle cells through a cGMP-independent mechanism [24].

In pregnant women, low respiratory CO levels are associated with hypertension in pregnancy and preeclampsia [43]. CO has been shown to induce vasodilation of human placental resistance blood vessels via activation of sGC in vitro [44]. Additionally, deficiencies in HO-1 impair placenta development which have been associated with pregnancy disorders, such as recurrent miscarriages, IUGR, and preeclampsia [45].

### 2.3. Hydrogen Sulfide

Figure 1 illustrates major enzymes for H_2_S synthesis, including cystathionine β-synthase (CBS), cystathionine γ-lyase (CSE), and 3-mercaptopyruvate sulfurtransferase (3MST) [6]. In the human placenta, these three enzymes are able to yield H_2_S [46]. CBS and CSE are cytosolic enzymes, but 3MST exists primarily in the mitochondria. Both CBS and CSE use L-Cysteine to generate H_2_S. In an alternative pathway, 3-mercaptopyruvate (3-MP), the substrate for 3MST to produce H_2_S, is provided by cysteine aminotransferase (CAT) and D-amino acid oxidase (DAO) [47]. In the peroxisome, D-Cysteine can be catabolized by DAO to generate H_2_S [17]. In addition to the enzymatic pathway, H_2_S can be produced via non-enzymatical pathway or by bacteria [48].

Uterine CBS and CSE levels increase during pregnancy and decrease during labor [24]. Like BK_Ca_, uterine smooth muscle K_ATP_ channel is important for uterine quiescence [49,50]. In humans, H_2_S can mediate vasodilation via K_ATP_ channel to maintain feto-placental circulation [51]. During pregnancy, CBS and K_ATP_ levels increase in human uterine artery smooth muscle cells [52]. However, decreased maternal H_2_S level and placental CBS and CSE protein levels relate to preeclampsia [51,53,54].

Collectively, NO, CO, and H_2_S play crucial roles for normal pregnancy. Dysregulated gasotransmitter signaling has been linked to preeclampsia, IUGR, stillbirth, and preterm labor [24,25]. Although specific mechanisms mediating cellular and organismal changes in pregnancy due to gasotransmitters await further exploration, emerging evidences suggest their therapeutic potential for compromised pregnancy to improve maternal and fetal outcomes.

## 3. Implications of Gasotransmitters in Hypertension and Kidney Disease

### 3.1. Gasotransmitters and Hypertension

Several lines of evidence indicate that NO, CO, and H_2_S play key roles in the pathogenesis of hypertension. The first are observations on knockout mice lacking genes responsible for gasotransmitter synthesis. First, eNOS knockout mice displayed hypertension [55]. The importance of H_2_S-generating enzymes in hypertension has also been demonstrated using CSE, CBS, or 3MST knockout mice [47,56,57,58]. Another report showed male HO-2 knockout mice are prone to develop renovascular hypertension [59].

The second line of evidences report dysregulated gasotransmitter signaling pathways in human and experimental models of hypertension. Prior research has addressed impaired L-Arginine–ADMA–NO pathway in the development of hypertension [60]. Dysregulated HO-1–CO pathway was reported to induce vascular dysfunction and hypertension in various animal models [61]. Likewise, deficiencies in H_2_S-generating enzymes and/or activity in hypertension has been established in various animal models, including the NO-deficient rats [62], the Dahl salt-sensitive rats [63], the spontaneously hypertensive rat (SHR) [64], and the renovascular hypertensive model [65].

Third, several therapeutic strategies targeting different gasotransmitters have demonstrated to be significant promising for beneficial effects against hypertension in various animal models [60,61,66,67,68].

### 3.2. Gasotransmitters and Kidney Disease

The gasotransmitter generating enzymes iNOS, eNOS, nNOS, HO-1, HO-2, CSE, CBS, and 3MST were detected in kidney cells comprising podocytes, glomerular endothelial cells, tubular cells, and mesangial cells, but not all of them are constitutively expressed in every cell type [69,70]. For example, eNOS is expressed in the glomerular endothelial cells, peritubular capillaries, and vascular bundles, while nNOS is mainly detected in the tubular epithelial cells of the macula densa [70]. Of note, iNOS and HO-1 are not constitutively expressed in the kidney but only expressed under certain pathophysiological conditions like inflammation [70].

In the kidney, NO performs important signaling functions including the modulation of renal sympathetic neural activity, control of renal hemodynamics, regulation of pressure-natriuresis, blunting of tubuloglomerular feedback, and inhibition of tubular sodium reabsorption [70]. Accordingly, impaired NO signaling has been implicated in the pathogenesis of kidney diseases. As reviewed elsewhere [14,71], kidney injury is attributed to NO deficiency in a variety of CKD models, such as diabetic nephropathy, chronic glomerular nephritis, the 5/6 nephrectomy model, the aging kidney, the Zucker obese rat, chronic allograft nephropathy, etc.

The beneficial actions of CO in the kidney have also been recognized [15]. Inhibition of superoxide production, activation of sGC, stimulation of NO production, and stimulation of p38 mitogen-activated protein kinase (MAPK) pathway are all examples of the beneficial effects of CO in the kidney to protect the kidney [15]. Deficiency or inhibition of HO-1 in animal models worsens renal structure and function, while increased expression is protective [72]. So far, evidences from animal models indicate that several kidney diseases have been associated with impaired HO-1 or -2 system, including diabetic nephropathy [73], lupus nephritis [74], nephrotoxic nephritis [75], ischemia-reperfusion injury [76], obstructive nephropathy [77], and CKD [78].

H_2_S regulates basic physiologic mechanisms of the kidney such as sodium reabsorption, glomerular filtration, and renal homeostasis [17]. In some animal models of kidney disease, such as CKD [79], acute kidney injury [80], cisplatin nephropathy [81], obstructive nephropathy [82], and diabetic nephropathy [83], it can serve as an agent that ameliorates kidney injury.

### 3.3. Crosstalk between NO, CO, and H_2_S in the Kidney and BP Control

Although NO and H_2_S share the same sGC–cGMP pathway to elicit relaxation in kidney cells [16], they act at different levels, with NO increasing production of cGMP through stimulation of sGC and H_2_S inhibiting cGMP degradation [84]. In rats, inhibition of NO by L-NAME, causes hypertension that can be prevented by the administration of sodium hydrosulfide (NaHS, a H_2_S donor), which also rescues NO bioavailability [85]. These data support the notion that there exists a NO/H_2_S crosstalk in the control of blood pressure (BP).

One of the NO-based cellular signaling pathways is via protein S-nitrosylation, the covalent addition of NO moiety to the sulfur atom of cysteine residues [86]. S-nitrosylation of specific proteins has been shown to be protective against kidney injury [86]. As observed for NO, H_2_S also employs post-translational modifications, namely, S-sulfhydration [87]. Endogenous H_2_S physiologically S-sulfydrates proteins on the thiol group of cysteine residues (e.g., glutathione), leading to the formation of the –SSH moiety. These observations lead to a hypothesis that there might be competition between S-nitrosylation and S-sulfhydration for the same cysteine residues in proteins, thus allowing the two gasotransmitters to regulate each other [84].

CO could also target sGC and regulate NO-mediated vasodilatation [88], which was supported by a report showing that transgenic mice overexpressing cell-specific HO-1 exhibit hypertension coinciding with decreased cGMP production in response to NO [89]. Additionally, CO could interfere with NOS activity and reduce NO generation as a consequence, thereby limiting NO-mediated vasodilation [90]. Although much is known about the CO and NO signaling pathways in the kidney, we so far do not fully understand how these two gaseous signaling systems interact with each other.

**Figure 2:**
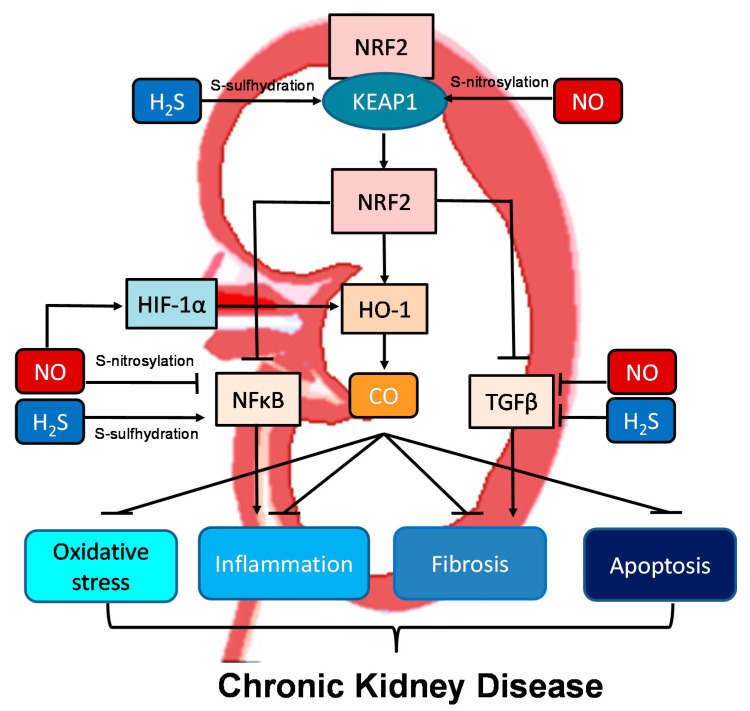
Schematic illustration of the crosstalk among NO, CO, and H_2_S and downstream nuclear factor erythroid 2-related factor 2 (NRF2)–heme oxygenase-1 (HO-1) signaling able to protect chronic kidney disease progression. Activation of the NRF2–HO-1–CO signaling pathway suppresses nuclear factor κB (NFκB) and transforming growth factor-β (TGF-β), consequently inhibiting oxidative stress, inflammation, fibrosis, and apoptosis. NO and H_2_S can activate NRF2 via S-nitrosylation and S-sulfhydration of Kelch-like ECH associated protein 1 (KEAP1), respectively. Via S-nitrosylation, NO can also inhibit NFκB-induced inflammation and activate hypoxia-inducible factor-1α (HIF-1α), a HO-1 inducer. Additionally, NFκB can also be regulated by H_2_S via S-sulfhydration. NO and H_2_S also reduce TGFβ-mediated fibrosis.

Moreover, all three gasotransmitters are involved in activation of nuclear factor erythroid 2-related factor 2 (NRF2) (Figure 2). NRF2 is a major regulator of HO-1 transcription responding to oxidative stress [72]. Upon activation, the NRF2-HO-1 pathway protects chronic kidney disease progression related to reduction of oxidative stress, inhibition of transforming growth factor-β (TGF-β)-driven fibrosis, reduction of inflammation and apoptosis [91]. Under basal conditions, NRF2 levels are kept low through the interaction with Kelch-like ECH associated protein 1 (KEAP1). Upon binding, the NRF2-KEAP1 interaction stabilizes the complex allowing for ubiquitylation, and ultimately proteasomal degradation of NRF2 [91]. Of note, NO and H_2_S can activate NRF2 via S-nitrosylation and S-sulfhydration of KEAP1, respectively [92,93]. In addition to NRF2, NO can regulate other redox-regulatory transcription factors, like nuclear factor κB (NFκB) and hypoxia-inducible factor-1α (HIF-1α), via S-nitrosylation [92]. As NFκB mediates inflammation and HIF-1α induces HO-1 expression, NO can interact with the NRF2–HO-1–CO signaling pathway in many different ways to prevent CKD progression. NFκB can also be S-sulfhydrated by H_2_S [93]. These observations indicate crosstalk mechanisms between NO, CO, and H_2_S are important determinants for kidney disease (Figure 2).

Although the beneficial actions of gasotransmitters against established kidney disease and hypertension have been established, their roles in mediating programmed responses behind developmental origins remain unclear. For this reason, this review will next outline the potential early-life interventions targeting NO, CO, and H_2_S signaling that may pose new opportunities for the therapeutic protection of hypertension and kidney disease.

## 4. Developmental Origins of Hypertension and Kidney Disease

### 4.1. Animal Models of Gasotransmitter-Related Renal Programming

So far, little reliable information exists regarding the impact of gasotransmitters on the development of hypertension and kidney disease in humans. Animal models enable researchers to consider various adverse environmental conditions in developmental stages to determine underlying programming processes and long-term outcome in adult offspring.

The developing kidney is extremely vulnerable to the effects of adverse environmental events, resulting in renal programming and eventually functional alterations and structural changes [94]. As reviewed elsewhere [10,12,21,94,95], renal programming is the major determinant of hypertension and kidney disease of developmental origins. Animal models particularly have provided more direct insight into the association between NO, CO, H_2_S, and renal programming. The current review is solely restricted to early-life insults starting in pregnancy and lactation period with focusing on gasotransmitter-related renal programming. Table 2 illustrates a variety of adverse conditions during pregnancy and lactation which may affect kidney development, resulting in hypertension and adverse renal outcomes in adulthood [71,96,97,98,99,100,101,102,103,104,105,106,107,108].

As shown in Table 2, the most common phenotype of renal programming being studied is hypertension [71,96,97,98,99,100,101,102,103,104,105,106,107,108]. Reduced nephron number has been demonstrated in offspring rats born of dams with caloric restriction [96,97] or streptozotocin-induced diabetes [98].

Our previous study reported that ADMA (a reactive oxygen species (ROS) inducer and endogenous NOS inhibitor) impaired ureteric bud branching morphogenesis, consequently leading to decreases of nephron number [32]. Additionally, kidney injury was addressed in models of maternal caloric restriction [96,97] and streptozotocin-induced diabetes [98,106]. Renal function was not determined or unaltered in most models of renal programming. In one study, GFR was decreased in 20-week-old mice offspring born to dams developed streptozotocin-induced diabetes [106]. Our review implicates that various early-life insults are relevant to renal programming, including maternal nutritional imbalance [96,97,100,108], maternal illnesses [98,99,101,106], prenatal environmental chemical exposures [104,105], and medication use during pregnancy [102,103,104].

Most studies focused on gasotransmitter NO [71,96,97,98,99,100,101,102,103,104,105], followed by H_2_S [99,103,107,108] and CO [106]. Impaired ADMA–NO pathway was reported in several models of renal programming, including maternal caloric restriction [96,97], streptozotocin-induced diabetes [98], maternal suramin administration [99], high-fructose diet [100], maternal adenine-induced CKD [101], prenatal glucocorticoid exposure [71,102], prenatal dexamethasone plus high-fat diet [103], prenatal dexamethasone plus TCDD exposure [104], and combined bisphenol A and high-fat diet exposure [105].

However, there is only one report demonstrating HO-1 is involved in maternal diabetes-induced hypertension and kidney disease [106]. Moreover, reduced renal H_2_S-synthesing enzyme expression, decreased renal H_2_S releasing activity, and low plasma H_2_S level have been reported in various models of programmed hypertension [99,103,107,108].

### 4.2. Therapeutic Prevention of Gasotransmitters for Hypertension and Kidney Disease of Developmental Origins

Various early-life insults can cause similar renal phenotypes, implying the existence of common pathways behind renal programming that may contribute to hypertension and kidney disease of developmental origins. Although the pathogenetic mechanisms have not yet been fully disclosed, certain renal programming mechanisms have been documented, including but not limited to, oxidative stress, aberrant renin–angiotensin system (RAS), dysregulated nutrient sensing signals, epigenetic regulation, gut microbiota dysbiosis, and sex differences [10,12,13,18,20,21,22,109,110,111]. Of note, each of the three gas signaling molecules has approximately mutual relationships with the above-mentioned mechanisms.

With a greater understanding of mechanisms behind renal programming, implementation of interventions for therapeutic prevention of hypertension and kidney disease in later life is feasible. An important message is that whereas therapeutic interventions can be delivered at any disease stage, reprogramming is barely restricted to key periods during early development. Here, we summarize the knowledge available today regarding gasotransmitters used as reprogramming strategies for developmental hypertension and kidney disease in various animal models [39,40,96,98,99,102,103,104,108,112,113,114,115,116,117,118,119,120,121], all of which are documented in Table 3. This review is only limited to gasotransmitter-based interventions as reprogramming strategies applied during pregnancy and/or lactation which are critical periods for kidney development.

Evidence from the studies reviewed indicates that rats are the most commonly used animal models. Rats become sexually mature at 6 weeks. In adulthood, one rat month is comparable to three human years [122]. Accordingly, Table 3 lists the therapeutic effects determined in rats ranging from 12 weeks to 8 months of rat age, which allows calculations to extract data for the specific age group that can be translated to humans. Note that little information currently exists in regard to large animals used for studying the roles of gasotransmitters on hypertension and kidney disease of developmental origins.

### 4.3. Nitric Oxide

Several therapeutic interventions have been used to increase NO bioavailability, such as supplementation of NO substrate, NO donors, ADMA-lowering agents, and enhancement of the expression and/or activity of NOS [18]. Nevertheless, only some of them have been reported for therapeutic prevention of programmed kidney disease and hypertension (Table 3).

L-Arginine supplementation has been considered as a therapeutic approach to improve NO bioavailability in human diseases [123], whereas its benefits from human trials remain inconclusive [124]. Although perinatal arginine supplementation combined with antioxidants has been reported to protect adult offspring against hypertension in spontaneously hypertensive rats (SHRs) and Fawn-hooded hypertensive (FHH) rats [125,126], whether perinatal arginine supplementation alone is able to reprogram hypertension and kidney disease of developmental origins has not been elucidated yet.

Because L-Citrulline can be converted to L-Arginine and it can bypass hepatic metabolism, oral L-Citrulline supplementation has been used as an add-on therapy to increase L-Arginine concentrations, subsequently increasing NO production [127]. Table 3 illustrates several models have been used to examine the reprogramming effects of perinatal L-Citrulline supplementation, including maternal N^G^-nitro–L-arginine methyl ester (L-NAME) exposure [40], maternal caloric restriction [96], streptozotocin-induced diabetes [98], and prenatal dexamethasone exposure [102]. In addition, early supplementation with L-Citrulline in young SHRs prevents the transition from prehypertension to hypertension [112].

The use of NO donors is another way to increase NO. Two NO donors—pentaerythritol tetranitrate and molsidomine—have been reported to prevent the development of hypertension in SHRs and FFH rats, respectively [113,114] (Table 3). However, so far little information exists with regard to NO donors in programming models to prevent kidney disease of developmental origins. Currently, a specific ADMA-lowering agent remains inaccessible. However, many currently used drugs have been reported to lower ADMA levels and restore NO bioavailability in human and experimental studies [14,19,106]. Among them—rosuvastatin, telmisartan, glucagon-like peptide-1 receptor agonist, and epigallocatechin-3-gallate—can decrease PRMT-1 (ADMA-generating enzyme) expression to reduce ADMA levels. Furthermore, telmisartan, resveratrol, metformin, melatonin, atorvastatin, N-acetylcysteine (NAC), vitamin E, salvianolic acid A, oxymatrine, and rosuvastatin have been reported to reduce ADMA level via enhancing the activity and/or expression of DDAHs (ADMA-metabolizing enzymes) [14]. Table 3 shows only few ADMA-lowering agents have been examined in the developmental programming models to prevent hypertension, including NAC [103], resveratrol [104], melatonin [115], and aliskiren [116]. Moreover, in mother rats that received melinjo (Gnetum gnemon) seed extract during lactation the development of hypertension programmed by excessive fructose intake in their female offspring could be prevented by enhancing eNOS expression [117].

### 4.4. Carbon Monoxide

As opposed to NO, limited information is available about the CO-based interventions to study their roles on kidney disease and hypertension of developmental origins. Carbon monoxide releasing molecules (CORMs), a group of chemical compounds capable of controlled CO release directly in tissues or organs, have emerged as a therapeutic tool for human diseases [128]. However, none of them have been examined in kidney disease and hypertension of developmental origins.

In addition to CORMs, HO-1 or Nrf2 activators are potential CO-based modalities to activate the NRF2–HO-1–CO signaling pathway. Many natural compounds have shown to be effective activators of NRF2/HO-1 like resveratrol, curcumin, quercetin, anthocyanins, carnosic acid, epigallocatechin gallate, celastrol, isothiocyanates, garlic-derived organosulfur compounds, etc. [129]. Aside from natural activators, some synthetic NRF2 activators have been developed for clinical application like dimethyl fumarate (DMF), oltipraz, and ursodiol [130].

There is general lack of studies investigating NRF2/HO-1 activators for the prevention of kidney disease of developmental origins. As shown in Table 3, only one study reported that maternal DMF treatment protected adult progeny against hypertension in a maternal dexamethasone exposure and postnatal high-fat diet model [118]. Our previous study demonstrated resveratrol therapy during pregnancy and lactation protects adult offspring against bisphenol A-induced liver damage is associated with activation of NRF2 [131]. Nevertheless, the impact of early-life resveratrol supplementation on NRF2/HO-1/CO signaling pathway awaits further elucidation.

### 4.5. Hydrogen Sulfide

So far, available H_2_S-based modalities used for therapeutic protection of hypertension and kidney disease include H_2_S donors, precursors of H_2_S, and organosulfur compounds. Inorganic sulfide salts like sodium hydrosulfide (NaHS) are the most widely used H_2_S donors to evaluate the therapeutic potential of exogenous H_2_S [68]. NaHS has shown anti-hypertensive effects in several hypertensive models, including NO-deficient rats [62], Dahl salt-sensitive rats [63], and SHRs [132]. In line with established hypertensive models, Table 3 shows maternal NaHS therapy during pregnancy and lactation periods prevented hypertension in adult offspring born to dams with renovascular hypertension [119]. Nevertheless, other H_2_S donors have not yet been tested in terms of their reprogramming effects on hypertension and kidney disease of developmental origins.

Precursors of H_2_S include L-Cysteine, D-Cysteine, and NAC, a stable cysteine analog. Table 3 shows perinatal NAC therapy protects adult offspring against hypertension programmed by various early-life insults, such as maternal L-NAME exposure [39], maternal suramin administration [99], prenatal dexamethasone and postnatal high-fat diet [103], maternal hypertension [108], and maternal nicotine exposure [120,121]. Although D- or L-cysteine supplementation between four and six weeks of age has been found to protect high salt-treated SHRs against hypertension and kidney injury at 12 weeks old [133], their uses in pregnancy and lactation implicating programming hypertension and kidney disease has not been explored yet. Another report demonstrated that supplementing garlic oil in pregnancy and lactation prevented hypertension programmed by a high-fat diet, which coinciding with increased expression and activity of H_2_S-producing enzymes in offspring kidneys [107]. Organosulfur compounds derived from garlic are natural precursors of H_2_S [134].

Several clinically used medications have been shown to increase H_2_S concentrations, such as amlodipine, aspirin, carvediol, atorvastatin, digoxin, paracetamol, metformin, ramipril, testosterone, vitamin D, and 17β-estradiol [135]. Additionally, significant progress has been achieved in recent years on new H_2_S-releasing drugs. It would be interesting to see whether these H_2_S-releasing drugs would appear to be a practical approach to prevent hypertension and kidney disease from further clinical translation.

Collectively, these findings indicate the potential impact of gasotransmitter-based interventions for therapeutic prevention of programmed kidney disease and hypertension. While these studies have also raised concern, the protective mechanisms behind some gasotransmitter-based interventions are not limited to only one gasotransmitter. For example, resveratrol has properties to lower ADMA and activate NRF2; however, to what extent its reprogramming effects on kidney disease and hypertension can be attributed to NO or CO deserves further clarification. Accordingly, a better understanding of each gasotransmitter-dependent and -independent mechanisms responsible for the reprogramming effects of various gasotransmitter-based interventions is therefore highly warranted.

## 5. Conclusions and Future Perspectives

Current evidences suggest a potential therapeutic role of gasotransmitter-based interventions for prevention of programmed hypertension and kidney disease. Although many NO-, CO-, and H_2_S-based drugs have led to a significant progress in our understanding of established hypertension and kidney disease, attention must be paid to prevent (and not just to treat) these diseases; translation from animal models into clinical practice will be an additional challenge.

Of note, much of the preclinical work investigated the reprogramming actions of NO and H_2_S, and most of them focused on hypertension of developmental origins. Nevertheless, there is little reliable information about the reprogramming effects of CO-based intervention. Meanwhile, we are aware that almost no studies have taken a holistic approach to simultaneous determinations of NO, CO, and H_2_S signaling pathway in one experiment. In view of the complex interplay between these three gasotransmitters, the reprogramming effect responding to each gasotransmitter-based intervention, either individually or in combination, are incomplete and difficult to predict. Furthermore, more attention should be paid to decide the optimal dosage and duration of gasotransmitter-based intervention using the appropriate animal models prior to clinical translation.

## Figures and Tables

**Table 1:** Physiological and toxic levels of NO, CO, and H_2_S.

	Physiological Level	Toxic Level	
Gas	Blood Concentration	Exposure Limit	IDLH
Nitric oxide (NO)	Low nM	TMA 25 ppm	100 ppm
Carbon monoxide (CO)	nM–μM	TMA 35 ppm/C 200 ppm	100 ppm
Hydrogen sulfide (H_2_S)	High nM–low μM	C 10 ppm	1200 ppm

TWA = Time-weighted average; C = Ceiling; IDLH = Immediately dangerous to life or health concentrations; ppm = parts per million.

**Table 2:** Summary of animal models of renal programming related to NO, CO, and H_2_S signaling.

Animal Models	Species/ Gender	Age at Evaluation	Mechanisms Related to Gasotransmitter	Renal Outcome and Blood Pressure	Ref.
Nitric oxide (NO)					
Maternal caloric restriction diet	SD rats/M	12 weeks	↑ ADMA ↓NO	Glomerular hypertrophy, ↑Tubulointerstitial injury and BP ↓Nephron number	[96,97]
Streptozotocin-induced diabetes	SD rats/M	12 weeks	↑ADMA ↓NO	↑Tubulointerstitial injury and BP ↓Nephron number	[98]
Maternal suramin administration	SD rats/M	12 weeks	↑ADMA ↓NO	↑BP	[99]
Maternal high-fructose diet	SD rats/M	12 weeks	↓NO	↑BP	[100]
Maternal adenine-induced CKD	SD rats/M	12 weeks	↑ADMA ↓NO	Renal hypertrophy ↑BP	[101]
Prenatal dexamethasone exposure	SD rats/M	16 weeks	↓Renal NO	↑BP	[102]
Prenatal dexamethasone exposure plus postnatal high-fat intake	SD rats/M	16 weeks	↓NO	↑BP	[103]
Prenatal dexamethasone plus TCDD exposure	SD rats/M	16 weeks	↑ADMA	↑BP	[104]
Prenatal bisphenol A exposure plus high-fat diet	SD rats/M	16 weeks	↑ADMA ↓NO	↑BP	[105]
Prenatal betamethasone exposure	Sheep/M and F	18 months	↓NO	↑BP	[71]
**Carbon monoxide (CO)**					
Streptozotocin-induced diabetes	Hoxb7-GFP-Tg mice/M	20 weeks	↑Renal HO-1 expression	Proteinuria, ↑Kidney injury, ↓GFR, ↑BP	[106]
**Hydrogen sulfide (H_2_S)**					
Maternal suramin administration	SD rats/M	12 weeks	↓Renal H_2_S releasing activity	↑BP	[99]
Maternal hypertension	SHRs/M	12 weeks	↓Renal 3MST protein expression & renal H_2_S releasing activity	↑BP	[107]
Prenatal dexamethasone exposure plus postnatal high-fat intake	SD rats/M	16 weeks	↓Renal CBS and 3MST protein expression	↑BP	[103]
Maternal and post-weaning high-fat diet	SD rats/M	16 weeks	↓Plasma H_2_S level	↑BP	[108]

Studies tabulated according to types of gasotransmitters, species, and age at evaluation. TCDD = 2,3,7,8-tetrachlorodibenzo-p-dioxin; CKD = Chronic kidney disease; ADMA = asymmetric dimethylarginine; SD = Sprague Dawley; SHR = spontaneously hypertensive rat; M = male; F = female; GFR = glomerular filtration rate; ↑ = increased; ↓ = decreased.

**Table 3:** Summary of gasotransmitter-based interventions used for therapeutic prevention of hypertension and kidney disease of developmental origins.

Gasotransmitter-Based Intervention	Animal Models	Species/ Gender	Age at Evaluation	Therapeutic Effects	Ref.
Nitric oxide (NO)					
Substrate for NOS					
0.25% L-Citrulline in drinking water during pregnancy and lactation	Maternal L-NAME exposure	SD rats/M	12 weeks	Prevented hypertension	[40]
0.25% L-Citrulline in drinking water during pregnancy and lactation	Maternal caloric restriction	SD rats/M	12 weeks	Prevented kidney damage, increased nephron number	[96]
0.25% L-Citrulline in drinking water during pregnancy and lactation	Streptozotocin-induced diabetes	SD rats/M	12 weeks	Prevented hypertension and kidney damage, increased nephron number	[98]
0.25% L-Citrulline in drinking water during pregnancy and lactation	Prenatal dexamethasone exposure	SD rats/M	12 weeks	Prevented hypertension, increased nephron number	[102]
L-Citrulline (2.5 g/L) in drinking water from 2 weeks before until 6 weeks after birth	Genetic hypertension model	SHR/M & F	50 weeks	Prevented hypertension	[112]
NO donors					
Pentaerythritol tetranitrate (50 mg/kg per day) during pregnancy and lactation	Genetic hypertension model	SHR/M & F	8 months	Prevented hypertension	[113]
Molsidomine (120 mg/L in drinking water) from 2 weeks before until 4 weeks after birth	Genetic hypertension model	FHH/M & F	42 weeks	Prevented hypertension	[114]
Asymmetric dimethylarginine (ADMA)-lowering agents					
Resveratrol (50mg/L in drinking water) during pregnancy and lactation	Prenatal dexamethasone plus TCDD exposure	SD rats/M	12 weeks	Prevented hypertension	[104]
Melatonin (0.01% in drinking water) during pregnancy and lactation	Maternal high-fructose diet plus post-weaning high-salt diet	SD rats/M	12 weeks	Prevented hypertension	[115]
Aliskiren (10 mg/kg/day) from 2 weeks to 4 weeks after birth	Maternal caloric restriction	SD rats/M	12 weeks	Prevented hypertension	[116]
NAC (1% in drinking water) during pregnancy and lactation	Prenatal dexamethasone exposure plus postnatal high-fat intake	SD rats/M	16 weeks	Prevented hypertension	[103]
Enhancement of NOS					
Melinjo (Gnetum gnemon) seed extract (1% in diet) from birth to postnatal week 3	Maternal high-fructose diet	Wistar rats/F	16 weeks	Prevented hypertension	[117]
**Carbon monoxide** **(CO)**					
NRF2 activator					
Daily oral gavage of dimethyl fumarate (50mg/kg/day) for 3 weeks during pregnancy	Prenatal dexamethasone exposure plus postnatal high-fat intake	SD rats/M & F	16 weeks	Prevented hypertension	[118]
**Hydrogen sulfide (H_2_S)**					
H_2_S donors					
Daily intraperitoneal injection of NaHS (56 μmol/kg/day) during pregnancy and lactation	2-kidney, 1-clip renovascular hypertension model	SD rats/M & F	16 weeks	Prevented hypertension	[119]
Precursors of H_2_S					
NAC (1% in drinking water) during pregnancy and lactation	Maternal L-NAME exposure	SD rats/M	12 weeks	Prevented hypertension	[39]
NAC (1% in drinking water) during pregnancy and lactation	Suramin administration	SD rats/M	12 weeks	Prevented hypertension	[99]
NAC (1% in drinking water) during pregnancy and lactation	Prenatal dexamethasone and postnatal high-fat diet	SD rats/M	12 weeks	Prevented hypertension	[103]
NAC (1% in drinking water) during pregnancy and lactation	Maternal hypertension	SHRs/M	12 weeks	Prevented hypertension	[108]
NAC (500 mg/kg/day) in drinking water from gestational day 4 to postnatal day 10	Maternal nicotine exposure	SD rats/M	8 months	Prevented hypertension	[120,121]
Organosulfur compounds					
Daily oral gavage of garlic oil (100 mg/kg/day) during pregnancy and lactation	Maternal and postweaning high-fat diet	SD rats/M	16 weeks	Prevented hypertension	[107]

Studies tabulated according to types of gasotransmitters and modalities, animal models and age at evaluation. L−NAME = N^G^-nitro-L-arginine-methyl ester. M = male. F = female. NAC = N-acetylcysteine. NaHS = sodium hydrosulfide. SHR = spontaneously hypertensive rat. SD = Sprague−Dawley rat. FHH = Fawn hooded hypertensive rat.

## Data Availability

Data will be available upon request.
